# Distribution and failure patterns of primary central nervous system lymphoma related to the hippocampus: implications for hippocampal avoidance irradiation

**DOI:** 10.1007/s11060-025-04965-7

**Published:** 2025-02-19

**Authors:** Hyejo Ryu, Xue Li, Tae Hoon Lee, Tae Min Kim, Seung Hong Choi, Chul-Kee Park, Soon Tae Lee, Sung-Hye Park, Jae-Kyung Won, Bum-Sup Jang, Il Han Kim, Joo Ho Lee

**Affiliations:** 1 Department of Radiation Oncology, Chung-Ang University Gwangmyeong Hospital, Gwangmyeong, Gyeonggii-Do Republic of Korea; 2 Department of Radiation Oncology, Seoul National University Hospital, Seoul National University College of Medicine, 101 Daehak-Ro, Jongno-Gu, Seoul, 03080 Republic of Korea; 3 Department of Radiation Oncology, The Affiliated Cancer Hospital of Zhengzhou University & Henan Cancer Hospital, Zhengzhou, People’s Republic of China; 4 Department of Radiation Oncology, Samsung Medical Center, Sungkyunkwan University School of Medicine, Seoul, Republic of Korea; 5 Department of Internal Medicine, Seoul National University Hospital, Seoul National University College of Medicine, Seoul, Republic of Korea; 6 Department of Radiology, Seoul National University Hospital, Seoul National University College of Medicine, Seoul, Republic of Korea; 7 Department of Neurosurgery, Seoul National University Hospital, Seoul National University College of Medicine, Seoul, Republic of Korea; 8 Department of Neurology, Seoul National University Hospital, Seoul National University College of Medicine, Seoul, Republic of Korea; 9 Department of Pathology, Seoul National University Hospital, Seoul National University College of Medicine, Seoul, Republic of Korea; 10 Cancer Research Institute, Seoul National University College of Medicine, Seoul, Republic of Korea; 11 Institute of Radiation Medicine, Medical Research Center, Seoul National University College of Medicine, Seoul, Republic of Korea

**Keywords:** PCNSL, Hippocampal avoidance, HA-WBRT, Consolidative RT

## Abstract

**Purpose:**

Hippocampal injury from WBRT contributes to neurocognitive decline in brain malignancy patients. HA-WBRT may mitigate this by reducing hippocampal radiation exposure, but its feasibility in PCNSL remains unassessed regarding hippocampal involvement and failure rates. This study evaluates hippocampal involvement at diagnosis and after treatment in PCNSL patients.

**Materials and methods:**

We conducted a retrospective analysis of 278 immunocompetent PCNSL patients diagnosed between 2000 and 2021. Following high-dose methotrexate-based induction chemotherapy, patients either received consolidation therapy, including RT, cytarabine alone, or autologous stem cell transplantation or underwent observation. Hippocampus was outlined on T1 MRI images and expanded by a 5 mm margin to create the hippocampal avoidance region (HAR). Hippocampal failure was defined as recurrence or progression at HAR. The median follow-up was 38.7 months (range 3.1–239.4 months).

**Results:**

Of the 278 patients diagnosed with PCNSL, 39.9% presented initial lesions at HAR. After induction therapy, 212 evaluable patients received consolidation treatments or observation. Intracranial failures occurred in 47.6% (n = 101), with 66.3% (n = 67) occurring outside the HAR and 33.7% (n = 34) inside the HAR. Unifocal disease (HR 0.61, 95% CI 0.39–0.96, p = 0.025) was associated with a lower risk of hippocampal failures, while initial HAR involvement significantly increased the risk (HR 2.26, 95% CI 1.18–4.47, p = 0.018). Patients with unifocal disease outside the HAR had the lowest 3-year hippocampal failure rate (6.2%). RT that included the hippocampus did not significantly affect hippocampal failure rates in patients without initial HAR lesions (p = 0.282), with three-year rates of 9.2 vs. 14.6% for other treatments. However, among patients with initial HAR involvement, RT including the hippocampus significantly reduced hippocampal failure rates compared to other approaches (p = 0.002). Hippocampal failure rates were comparable, with conventional WBRT at 14.6% and HA-WBRT at 19% in patients without initial HAR lesions (p = 0.734).

**Conclusion:**

The routine application of the HA-WBRT strategy is not supported due to the high risk of hippocampal failures in general and requires further investigation to establish its feasibility and safety in well-defined subgroups. Our results suggest that the HA-WBRT strategy could be evaluated for select PCNSL patients with unifocal lesions or those located outside the HAR.

**Supplementary Information:**

The online version contains supplementary material available at 10.1007/s11060-025-04965-7.

## Introduction

Primary central nervous system lymphoma (PCNSL) is a rare extranodal non-Hodgkin lymphoma, accounting for 4% of intracranial tumors. PCNSL management includes methotrexate-based induction therapy followed by various consolidation therapy options. Among them, consolidative whole brain radiotherapy (WBRT) plays a crucial role in preventing disease relapse, but its detrimental effects are significant. Long-term neurotoxicity post-treatment can severely impair the quality of life and functionality in PCNSL patients, particularly among the elderly [1, 2]. Consequently, whole brain radiation therapy should be judiciously considered in patients older than 60, though alternative consolidations such as autologous stem cell transplantation (ASCT) are generally not viable for older adults [3].

The neurotoxicity of conventional WBRT (C-WBRT) is closely linked to the hippocampus, and reducing radiation exposure to this area can help preserve memory functions. A preclinical model demonstrated dose-dependent damage to neural progenitor cells in the hippocampus [4]. Subsequently, several prospective clinical studies have confirmed the neurocognitive benefits of hippocampal avoidance during whole brain radiation therapy (HA-WBRT) in patients with brain metastases, prophylactic treatments, and more recently, primary brain tumors [5–7].

In this context, HA-WBRT emerges as a promising alternative treatment strategy that could maintain both tumor control and neurocognitive function, significantly enhancing the patient’s quality of life. Despite the urgency in preserving physical function for patients with PCNSL, limited data exists on the failure rate in the hippocampal area due to the disease’s rarity. Thus, the safety of hippocampal avoidance in PCNSL patients cannot be confidently assessed. In this study, we evaluated the risk of hippocampal area involvement at diagnosis and at recurrence in PCNSL patients.

## Materials and methods

### Study design and subject

This study was a single-institution retrospective study. The medical records at our institution from 2000 to 2021 were reviewed. The study received approval from the Seoul National Hospital’s Institutional Review Board (Number. H-2203-127-1308). The requirement for patient consent was waived due to the retrospective nature of the study. We included intracranial PCNSL patients with initial MRI images and histological diagnoses between January 2000 and December 2021. Patients with distant organ involvement other than the eyes and those with a history of intracranial radiation or systemic chemotherapy were excluded. A total of 278 patients were included to analyze the primary tumor location in relation to the hippocampus (Supplementary Table 1). Among the 278 patients diagnosed with PCNSL, 11 were unable to commence induction chemotherapy post-diagnosis due to poor performance status (Supplementary Fig. 1). A total of 10 patients were either transferred or lost to follow-up after their diagnosis, and one mortality was reported. Consequently, 256 patients began induction chemotherapy; however, 10 patients died during treatment, and 20 exhibited disease progression.

To ascertain the failure pattern, the study cohort was refined to include only those who received high-dose methotrexate-based chemotherapy without showing progression (refer to Supplementary Fig. 1). Patients lacking follow-up MRI data were excluded. Thus, a total of 212 patients were deemed eligible for failure pattern analysis in relation to the hippocampus. The baseline and treatment characteristics of this group are presented in Table 1. The median age at diagnosis was 61 (range, 24–85), and 141 patients (63.5%) had an ECOG performance status between 0 and 1. For diagnosis, brain stereotactic biopsy and surgery were performed in 185 (87.3%) and 27 (12.8%) patients, respectively. The predominant histology was diffuse large B cell type (DLBCL), representing 95.3% of the cohort. The remaining histology included T-cell type in 3 patients and other B-cell histologies in 7 patients. 24 patients (11.3%) had malignant cell-positive CSF cytology, and deep structure involvement including the basal ganglia, corpus callosum, brainstem, and cerebellum was observed in 135 patients (63.7%). Multifocality was classified based on the presence of multiple gross lesions identified on MRI. CSF cytology positivity was not considered in this classification or in determining multifocality and hippocampal involvement. Multifocal lesions were observed in 129 (60.8%) of the patients.

**Table 1:** Baseline characteristics of 212 PCNSL patients with induction therapy

Characteristics	N = 212 (Total)
Age at diagnosis (yr)	61 (24–85)
< 60	95 (44.8)
≥ 60	117 (55.2)
Sex	
Male	114 (53.8)
Female	98 (46.2)
ECOG performance status	
0–1	141 (63.5)
2–4	71 (33.5)
Pathology	
Diffuse large B-cell lymphoma	202 (95.3)
Other B-cell lymphoma	7 (3.3)
T-cell lymphoma	3 (1.4)
Eye involvement	
Yes	22 (10.4)
No	190 (89.6)
CSF cytology ( +)	
Yes	24 (11.3)
No	188 (88.7)
Diagnosis	
Stereotactic biopsy	185 (87.3)
Surgery	27 (12.8)
Number of lesions	
Single	83 (39.2)
Multiple	129 (60.8)
Deep structure involvement	
Yes	135 (63.7)
No	77 (36.3)
Chemotherapy regimens	
MTX+Rituximab	122 (57.5)
MTX—Rituximab	90 (42.5)
Total cycles	6 (1–8)
Induction treatment response	
Complete	152 (71.7)
Partial response	56 (26.4)
Stable disease	4 (1.9)
Consolidative Treatment	
Observation	38 (17.9)
RT and Cytarabine	71 (33.5)
RT only	74 (34.5)
Cytarabine only	24 (11.3)
Autologous stem cell transplant	5 (2.4)

Radiotherapy characteristics	N = 145
Field	
Conventional WBRT	114 (78.6)
Hippocampal avoidance whole brain a)	23 (15.9)
Focal	8 (5.5)
Dose	
RT, median (range)	27 Gy (18–45 Gy)
Total, median (range)	45 Gy (16.4–60 Gy)

Data are presented as number (%) or median (range)

*yr* Year; *MTX* Methotrexate; *RT* Radiotherapy; *WBRT* Whole Brain Radiotherapy; *Gy* Gray

### Treatment

As induction therapy, patients received a median of 6 cycles (range, 1–8) of high-dose methotrexate-based chemotherapy. Most patients (195) followed the RTOG 9310 protocol, receiving methotrexate, vincristine, and procarbazine (MVP) [8]. Alternative regimens included methotrexate, vincristine, and dexamethasone (MVD), and mitoxantrone, vincristine, and dexamethasone (MOD). Methotrexate alone was administered to one patient, and methotrexate combined with cytarabine to two patients. After 2014, Rituximab was added to the regimen and administered to 122 patients (57.5%) in combination with methotrexate, procarbazine, and vincristine (MVP). Intrathecal methotrexate was administered to 16 patients due to positive CSF cytology.

Post-induction therapy outcomes were as follows: Complete response (CR) in 152 patients (71.7%), partial response (PR) in 56 patients (26.4%), and stable disease (SD) in 4 patients (1.9%). The choice of consolidative therapy was based on clinical judgment, considering the patient’s age and performance status. Consolidative treatments included observation (n = 38, 17.9%), radiation and cytarabine (n = 71, 33.5%), radiation alone (n = 74, 34.5%), cytarabine alone (n = 24, 11.3%), and autologous stem cell transplant (n = 5, 2.4%). After consolidative treatments, 178 patients (84.0%) achieved CR during the follow-up duration.

Variations in the radiation fields were noted among 145 patients receiving consolidative RT. The choice of radiation field was based on clinical judgement, considering the disease status and the patient’s performance and concerns about neurocognitive function. The most common RT field was the whole brain including hippocampus, used in 114 patients (78.6%), followed by HA-WBRT in 23 patients (15.9%) and focal RT in 8 patients (5.5%). A sequential tumor bed boost followed WBRT. The median WBRT dose was 27 Gy (range 18–45 Gy) and the median total dose was 45 Gy (range 16.4–60 Gy). HA-WBRT was performed in the patients without initial HAR involvements, while 64.9% of patients receiving C-WBRT did not have initial HAR involvements.

### Endpoints and image analysis

Our primary endpoint was the incidence of hippocampal area involvement at both diagnosis and relapse. To measure the distance between the tumor and the hippocampus, we imported brain MRI images into our treatment planning system (Varian Eclipse). We manually contoured both the tumor and the hippocampus according to the RTOG 0933 protocol [9]. Subsequently, we created 3-dimensional expansions of the hippocampus by 5 mm and 15 mm. The hippocampal avoidance region (HAR) was defined as an area within 5 mm of the hippocampus. Hippocampal failure referred to relapse or progression within the HAR region on follow-up contrast-enhanced T1 MRI images. T2 MRI images were used to detect recurrent lesions not visible on T1 MRI images. Intracranial failure was defined as relapse or progression within the brain. Neurotoxicity was assessed through a retrospective review of medical records in the patients receiving consolidative radiotherapy. To eliminate the influence of disease-related effects, 117 patients who achieved complete response (CR) after consolidative radiotherapy were included in the analysis. Treatment-induced neurotoxicity was defined as the onset of grade 2 or higher cognitive dysfunction, memory loss, or dysarthria following the completion of consolidative treatments. The severity of neurotoxicity was evaluated using the Common Terminology Criteria for Adverse Events (CTCAE) version 5.0.

### Statistical analysis

Basic descriptive statistics for patient and treatment characteristics were presented as numbers and percentages, as well as medians with ranges and means with standard deviations. The Kaplan–Meier method was utilized to calculate hippocampal failure rate, progression-free survival (PFS), overall survival (OS). PFS was defined as the time from the date of initial pathological diagnosis to the date of either intracranial or extracranial progression or death from any cause. OS was defined as the time from the date of initial pathological diagnosis to the date of death from any cause. Hippocampal failure rate was measured from the initial pathological diagnosis to the occurrence of failures involving the HAR, with events censored at death or the last follow-up in the absence of hippocampal failure. To identify prognostic factors for hippocampal failure, we employed Cox regression analysis. P values less than 0.05 were deemed statistically significant. Statistical analyses were conducted using Stata 16.1 (StataCorp, College Station, TX) and Prism 10.2.0 (Graphpad Software).

## Results

### Location of primary tumors in relation to hippocampus

**Fig. 1:**
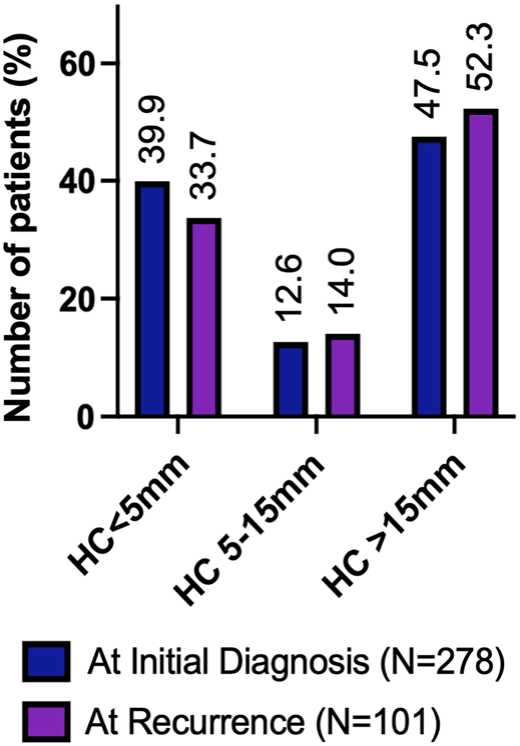
Distribution of PCNSL patients according to the lesions in relation to hippocampus at initial diagnosis and recurrence

In the initial cohort of 278 patients, 39.9% had primary tumors located within the HAR at diagnosis, while other 60.1% had primary tumors outside the HAR at diagnosis. There were 35 (12.6%) and 132 (47.5%) patients with initial tumors located 5–15 mm and more than 15 mm from the hippocampus, respectively, as shown in Fig. 1.

### Hippocampal failure incidence and failure pattern

The median follow-up period was 38.7 months (ranging from 3.1 to 239.4 months). The 3-year overall survival and PFS were 67.3% and 49.3%, respectively. The 3-year hippocampal failure rate was 17.0% (Supplementary Fig. 2). A total of 101 patients experienced intracranial failure, with 67 patients (66.3% of those with intracranial failure) having recurrent tumors outside the HAR (Fig. 1). Local failure was observed in 48 cases (47.5%), while distant failure occurred in 40 patients (39.6%). Additionally, 13 patients (12.9%) experienced relapses at both local and distant sites.

Unifocal disease (HR 0.61, 95% CI 0.39–0.96, p = 0.025) was associated with a lower risk of hippocampal failures, while initial HAR involvement significantly increased the risk (HR 2.26, 95% CI 1.18–4.47, p = 0.018) (Table 2). However, sex, age, surgery method, performance, CSF cytology, deep structure involvements, and the response to induction therapy were not associated with hippocampal failures.

**Table 2:** Risk factors for hippocampus failure in patients (N = 212)

	HR	95% CI	P value
Male (vs Female)	0.69	0.34–1.41	0.315
Age ≥ 60 (vs < 60)	1.07	0.54–2.13	0.837
Surgery (vs Biopsy)	0.89	0.40–1.97	0.784
ECOG 2–4 (vs. 0–1)	1.14	0.55–2.35	0.722
CSF cytology + (vs No)	1.56	0.67–3.58	0.297
Unifocal (vs Multifocal)	0.35	0.15–0.81	0.014
Deep structure + (vs No)	1.68	0.82–3.45	0.151
Initial involvement of HAR (HC < 5 mm)	2.26	1.18–4.47	0.018
CR after induction therapy (vs non-CR)	1.464	0.70–3.06	0.311

*ECOG* Eastern Cooperative Oncology Group; *CSF* Cerebrospinal Fluid; *HAR* Hippocampal Avoidance Region; *CR* Complete Response; *HR* Hazard Ratio; *CI* Confidence Interval

Focal RT to only gross lesion was adapted in 8 patients. Local failures were observed in two patients and distant failures in one patient. The 3-year overall survival and PFS in these patients were 62.5% and 62.5%, respectively.

### Stratification by subgroups

**Fig. 2:**
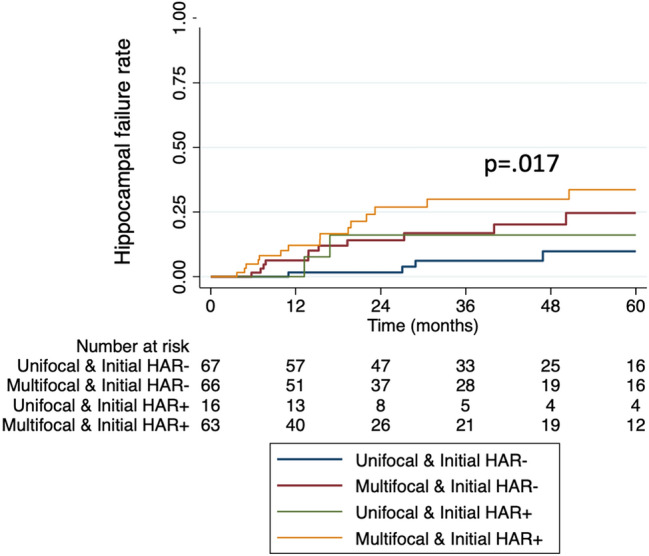
Kaplan–Meir curve of hippocampal failure rate in 212 PCNSL patients according to number of lesions and initial locations in relation to hippocampus

Patients were stratified into four groups based on initial HAR involvement and multifocality: Unifocal lesion without initial HAR involvement (n = 67), multifocal lesion without initial HAR involvement (n = 66), unifocal lesions with initial HAR involvement (n = 16), and multifocal lesions with HAR involvement (n = 63). The 3-year PFS rates were 61.1%, 49.6%, 43.6%, and 37.8% (p = 0.170), showing no statistically significant difference, while the cumulative 3-year hippocampus failure rates were 6.2%, 16.8%, 16.1%, and 26.9% (p = 0.017), indicating a statistically significant difference. (Fig. 2).

**Fig. 3:**
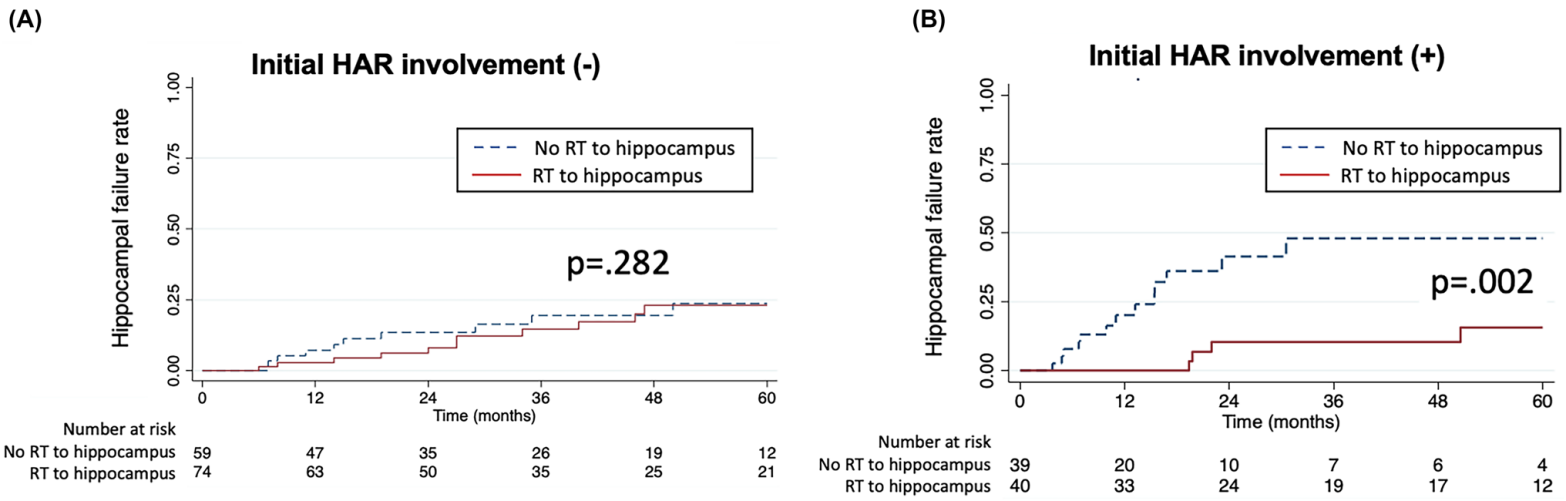
Kaplan–Meir curve hippocampal failure rate by RT to hippocampus without (**a**) and with (**b**) initial HAR involvements. The “No RT to hippocampus” group includes HA-WBRT, focal RT, no consolidation, chemotherapy only, ASCT

To assess the impact of RT targeting the hippocampus on hippocampal failure, we compared the hippocampal failure rates between RT to hippocampus and initial HAR involvements. RT to hippocampus was defined as homogeneous dose delivery to the entire brain including both hippocampal regions. In contrast, no RT to the hippocampus included all other approaches that did not involve hippocampal irradiation, such as HA-WBRT, focal radiation to the gross tumor, chemotherapy, and observation. Among 133 patients with unifocal or multifocal lesions outside HAR, RT to hippocampus showed no significant impact on hippocampus failure incidence with a 3-year rate of 14.6%, compared to a 19.4% rate in the no RT to hippocampus group (p = 0.282, Fig. 3A). Conversely, there was a significant difference in 3-year hippocampus failure incidence by C-WBRT in 79 patients with initial lesions involving HAR (10.4% vs 47.9%, P = 0.002, Fig. 3B).

**Fig. 4:**
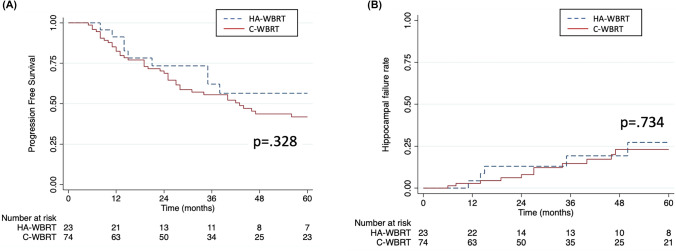
Kaplan–Meier curves comparing (**a**) progression-free survival and (**b**) hippocampal failure rates between C-WBRT and HA-WBRT in patients without initial HAR involvement

Additionally, progression-free survival (PFS) and hippocampal failure rates were analyzed in patients without initial HAR involvement, comparing C-WBRT and HA-WBRT. The 3-year PFS rates showed no significant difference between C-WBRT (56%) and HA-WBRT (62%) (p = 0.328, Fig. 4A). Similarly, hippocampal failure rates were comparable, with C-WBRT at 14.6% and HA-WBRT at 19% (p = 0.734, Fig. 4B). Following induction therapy, the proportions of CR, PR, and SD were 17 (74%), 6 (26%), and 0, respectively, in the HA-WBRT group, and 42 (57%), 31 (42%), and 1 (1%), respectively, in the C-WBRT group, with no significant difference observed between the two groups (p = 0.140).

### Neurotoxicity

Among the 117 patients who achieved CR following consolidative radiotherapy, 32 (27%) experienced treatment-related neurotoxicity during the follow-up period. Based on the radiotherapy field, neurotoxicity was observed in 1 patient (13%) receiving focal RT, 3 patients (14%) receiving HA-WBRT, and 28 patients (32%) receiving C-WBRT. Among patients without HAR involvement, neurotoxicity occurred in 14% (3 of 21) of those treated with HA-WBRT and 33% (19 of 58) of those treated with C-WBRT, though the difference did not reach statistical significance (p = 0.110).

## Discussion

To investigate region-specific targeting in radiotherapy for prospective trials, such as HA-WBRT, analyzing patterns of involvement and failures in conventional treatment groups will be essential [10–13]. This analysis can provide clinical evidence to support future prospective clinical trials, as shown in its applications in brain metastasis or prophylactic cranial irradiation for small cell lung cancer [14]. In retrospective data of initial and recurrent PCNSL, we found that at diagnosis, approximately 40% of patients had tumors within HAR, and 33.7% experienced recurrent tumors near the hippocampus. We identified that multifocality and initial HAR involvement were associated with an increased risk of hippocampal failure. The lowest hippocampal failure rates were observed in patients with unifocal lesions located away from the hippocampus. Additionally, the hippocampal failure rates in patients without initial HAR involvement remained unchanged in the group that did not receive RT to hippocampus.

Although methotrexate-based systemic treatment followed by consolidation has enhanced disease control, it has led to the emergence of delayed cognitive impairment, affecting up to 30% of PCNSL patients—especially the elderly—highlighting the need to weigh therapeutic efficacy against quality-of-life impacts [15, 16]. The deintensification strategy aimed at minimizing neurotoxicity risk in PCNSL patients focuses on reducing radiation dosage. Consequently, consolidative WBRT using a reduced dose has supplanted the standard dose. Morris et al.’s multicenter phase II study was the initial study to report excellent overall survival and tolerable toxicity with reduced dose WBRT and cytarabine as consolidation therapy in patients achieving CR after R-MPV induction treatment [17]. Preliminary results from RTOG 1114 further confirmed similar findings [18]. Additionally, a large national cohort study in France observed improved cognitive function in those treated with a reduced WBRT dose [19]. Similarly, our previous study indicated that reduced dose WBRT offered comparable tumor control with less neurotoxicity than the standard dose [20].

Contrary to the success of the dose-reduction strategy, using focal irradiation instead of whole-brain techniques did not yield satisfactory tumor control or acceptable toxicity levels. Shibamoto et al.’s retrospective studies reported high recurrences rates both infield and outfield, at 57% and 49% respectively [21]. Although our study reported more favorable outcomes with a 25% rate of local failure and a 12.5% rate of distant failure, our small sample size should be taken into account. Similarly, doubts have arisen regarding the replacement of WBRT with stereotactic radiosurgery (SRS). Despite its high biological effective dose and the potential to prevent cognitive decline, the concern about distant failures inhibits its broader use among clinicians. Hironoet al. observed distant failure in 75% of patients who received SRS following a high dose of MTX [22].

Given the close relationship between the hippocampus and neurocognition, the hippocampal avoidance technique may benefit patients with PNCSL by preserving neurological function [23]. Recently, the adoption of this technique in cranial radiation has expanded significantly. It is now routinely used in treating brain metastases or administering prophylactic cranial irradiation. Specifically, conventional WBRT has been associated with neurocognitive deficits, including impairments in memory, attention, and executive function, all of which relate to hippocampal activity [24, 25]. In this study, HA-WBRT exhibited lower percentage of treatment-related neurotoxicities, although it did not reach statically significant difference from C-WBRT due to the small number of patients.

Until now, the use of HA-WBRT in patients with PCNSL remains controversial, with only a few studies investigating its feasibility. One such study is a case report advocating for HA-WBRT. In a recent report by Yeo et al., two patients with a single tumor located away from the hippocampus underwent R-MPV induction chemotherapy followed by hippocampal-avoidance bilateral WBRT [26]. Over follow-ups of 51 months and 19 months, these two patients exhibited neither tumor recurrence nor neurological toxicity, demonstrating that HA-WBRT may effectively preserve neurological function and control tumor growth despite the limited sample size. Moreover, Mazzarella et al. analyzed 43 PCNSL patients with 66 lesions and found that 30.3% of original lesions were located within the HAR, consistent with our findings. Post-WBRT, 16 out of 35 recurrences (45.7%) occurred within HAR [27]. Contrastingly, 11.1% of primary lesions located 15 mm from the hippocampus relapsed at HAR. Despite the lack of detailed systemic therapy information, a 45.7% hippocampal failure rate is notably high.

From a recurrence perspective, the use of HA-WBRT in PCNSL warrants cautious consideration, particularly for patients with initial HAR involvement, even those who achieve CR following treatment. The hippocampus is a distinctive neurogenic niche within the CNS, housing neural stem cells (NSCs), and emerging evidence suggests that NSCs may also act as a potential origin and reservoir for certain brain tumors [28–30]. Furthermore, tumor cells may preferentially target NSC-enriched regions, such as the hippocampus, due to the favorable conditions provided by these niches [31, 32]. Given these potentially tumorigenic properties, minimal residual disease may persist within the HAR despite imaging studies indicating CR [33]. These insights underscore the need to better understand the relationship between the hippocampus and recurrence in PCNSL. Future studies should explore the role of NSC niches in tumor recurrence and establish clear contraindications for the application of HA-WBRT. In our study, we observed a relatively high recurrence rate (34%) within the hippocampus among our PCNSL patients compared to those with brain metastases, where hippocampal failure rates are generally reported to be below 10% [34]. The routine application of the HA-WBRT strategy is not supported due to the high risk of hippocampal failures in general.

Given the relatively higher prevalence of hippocampal involvement in PCNSL, a unilateral hippocampal avoidance strategy could broaden the eligibility for HA-WBRT among PCNSL patients. Avoiding the hippocampus in the dominant hemisphere may be sufficient to preserve neurocognitive function while maintaining effective treatment outcomes [35]. Future studies should explore the feasibility and impact of unilateral hippocampal avoidance on neurocognitive preservation, which could contribute to further optimizing hippocampal avoidance radiotherapy.

Consequently, further research on the suitable candidates for HA-WBRT is crucial, and HA-WBRT should be evaluated in select cases. Our study may provide potential indications for HA-WBRT in PCNSL. Firstly, HA-WBRT could be suitable for lesions located outside of HAR. Patients with unifocal lesions appear to have a lower risk, making them the most suitable group for attempting HA-WBRT. Secondly, implementing HA-WBRT is advisable for elderly patients highly susceptible to neurocognitive decline. Age is significantly associated with neurotoxicity in PCNSL patients [15, 16, 20]. Elderly over age 60 experience neurotoxicity in about 30–40% after treatment, likely exacerbated by reduced cognitive reserve and diminished neuroplasticity. Especially, alternative standard consolidations such as autologous stem cell transplantation (ASCT) are generally not viable for older adults [3]. This highlights the need for strategies beyond merely reducing radiation dose to the whole brain. Preserving hippocampal integrity through HA-WBRT may not only mitigate neurotoxicity but also help maintain quality of life, including memory, attention, and executive functions, which are vital for independent living in this population. Given the rising incidence of PCNSL among the elderly and the limited evidence on HA-WBRT in this demographic, multicenter prospective trials are needed to establish optimized protocols.

There are limitations in our study. Firstly, the patient population was diverse, which can primarily be attributed to evolving PCNSL treatment strategies over the years. The study included patients before the advent of Rituximab. Additionally, the patients underwent various types of consolidation treatments, such as chemotherapy, ASCT, radiation therapy, and observation. We encompassed all forms of consolidation treatments to study the natural progression of the disease, particularly in cases where no radiation was administered to the hippocampus. Despite the variation in treatment regimens, the treatment strategy remained consistently applied across our single-institution data over the decades. Secondly, the retrospective, single-institution design of this study represents a key limitation, restricting the generalizability of our findings to broader clinical settings. The retrospective nature precludes the ability to control for certain confounding variables or ensure uniformity in data collection. Another concern is that HA-WBRT may pose a risk of early recurrence, not only in the hippocampal region but also in other distant areas. However, due to the heterogeneity of the study population and its retrospective design, accurately assessing this risk was limited. To overcome these limitations, multicenter prospective trials are essential. These studies should include precisely defined inclusion criteria to include potential low-risk candidates such as no HAR involvements or unifocal lesion. Moreover, standardized treatment protocols that clearly specify radiation dose, planning techniques of HA-WBRT, and delivery methods will be critical in minimizing variability and ensuring consistent treatment quality across study sites. In addition, objective and comprehensive neurocognitive assessments should be integrated into these trials to evaluate the true impact of hippocampal avoidance on preserving cognitive function. Such assessments, ideally conducted longitudinally, would provide robust evidence to support the neuroprotective benefits of HA-WBRT while balancing oncologic outcomes. By incorporating these elements, prospective studies can address the current gaps in evidence and help establish HA-WBRT as a viable treatment strategy in well-defined patient subgroups. Thirdly, this study’s limitations include the inability to fully assess the risks posed by CSF cytology positivity to hippocampal failures, due to the small number of patients in this subgroup. CSF cytology positivity is often associated with a more disseminated disease state, potentially posing an increased risk for treatment failures [36]. Until further studies provide a clearer understanding of the risks associated with CSF cytology positivity, caution would be exercised when considering HA-WBRT for these patients.

## Conclusion

HA-WBRT may present a viable treatment option for PCNSL patients to protect neurocognitive function. However, the routine application of the HA-WBRT strategy is not supported due to the high risk of hippocampal failures in general and requires further investigation to establish its feasibility and safety in well-defined subgroups. Our results suggest that the HA-WBRT strategy could be evaluated for select PCNSL patients with unifocal lesions or those located outside the HAR. Prospective studies are necessary to further evaluate the feasibility and effectiveness of HA-WBRT, particularly in subgroups at low risk for hippocampal failure who may benefit from neurocognitive protection.

## Supplementary Information

Below is the link to the electronic supplementary material.

Supplementary file1 (TIFF 5243 KB) Patient selection

Supplementary file2 (TIFF 5243 KB) Kaplan–Meir curve of overall survival (**a**), progression free survival (*b*), and hippocampal failure rate (**c**) in 212 PCNSL patients with induction chemotherapy

Supplementary file3 (TIFF 5243 KB) Summary of the key findings

Supplementary file4 (DOCX 21 KB)

## Data Availability

No datasets were generated or analysed during the current study.
