# Supplementary figures and images for: Distribution and failure patterns of primary central nervous system lymphoma related to the hippocampus: implications for hippocampal avoidance irradiation
Source: J Neurooncol. 2025 Feb 19;173(1):95–104. doi: 10.1007/s11060-025-04965-7 (PMC12041158; doi:10.1007/s11060-025-04965-7)

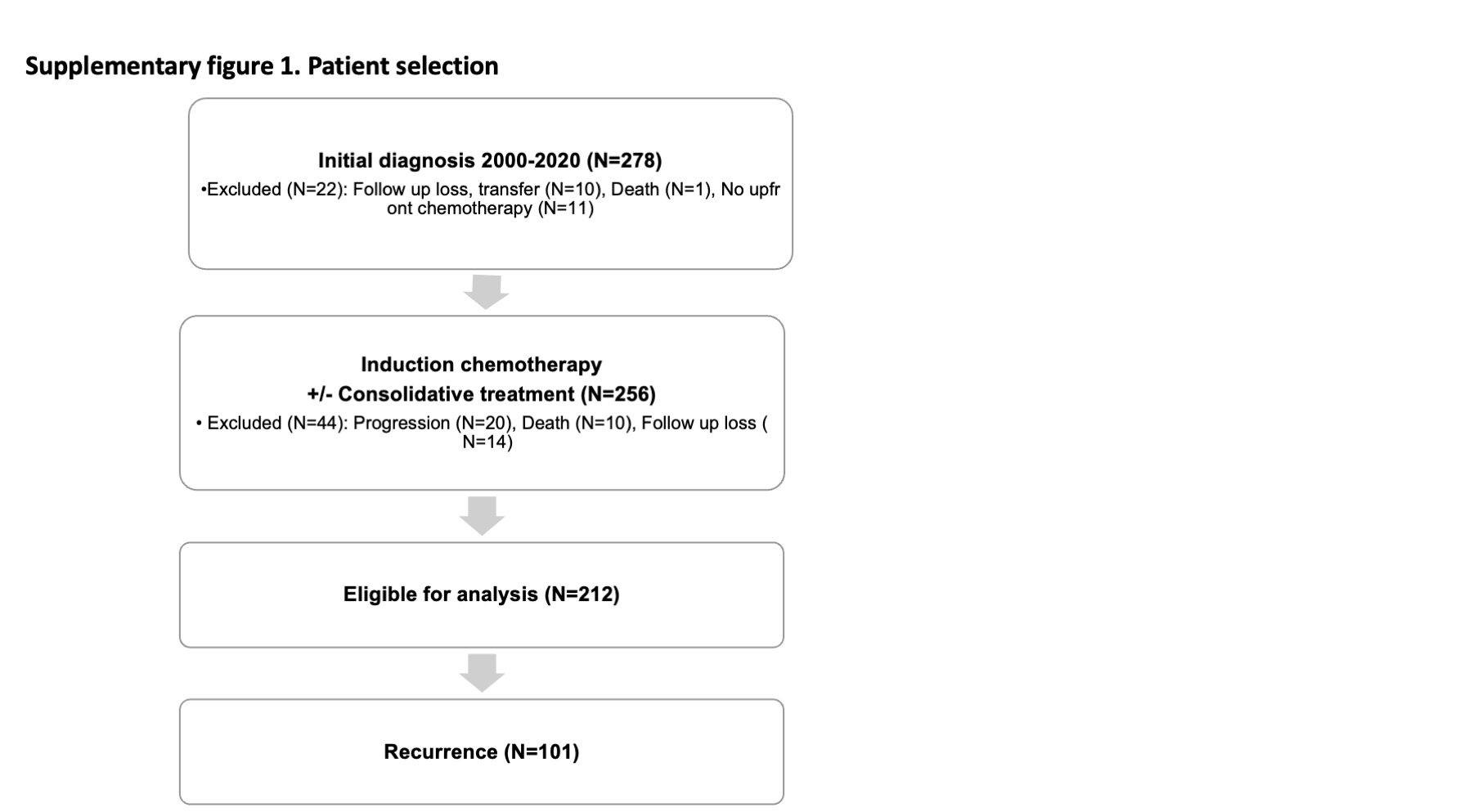

Supplement: Supplementary file 1 — Supplementary file1 (TIFF 5243 KB) Patient selection [file 11060_2025_4965_MOESM1_ESM.tiff]

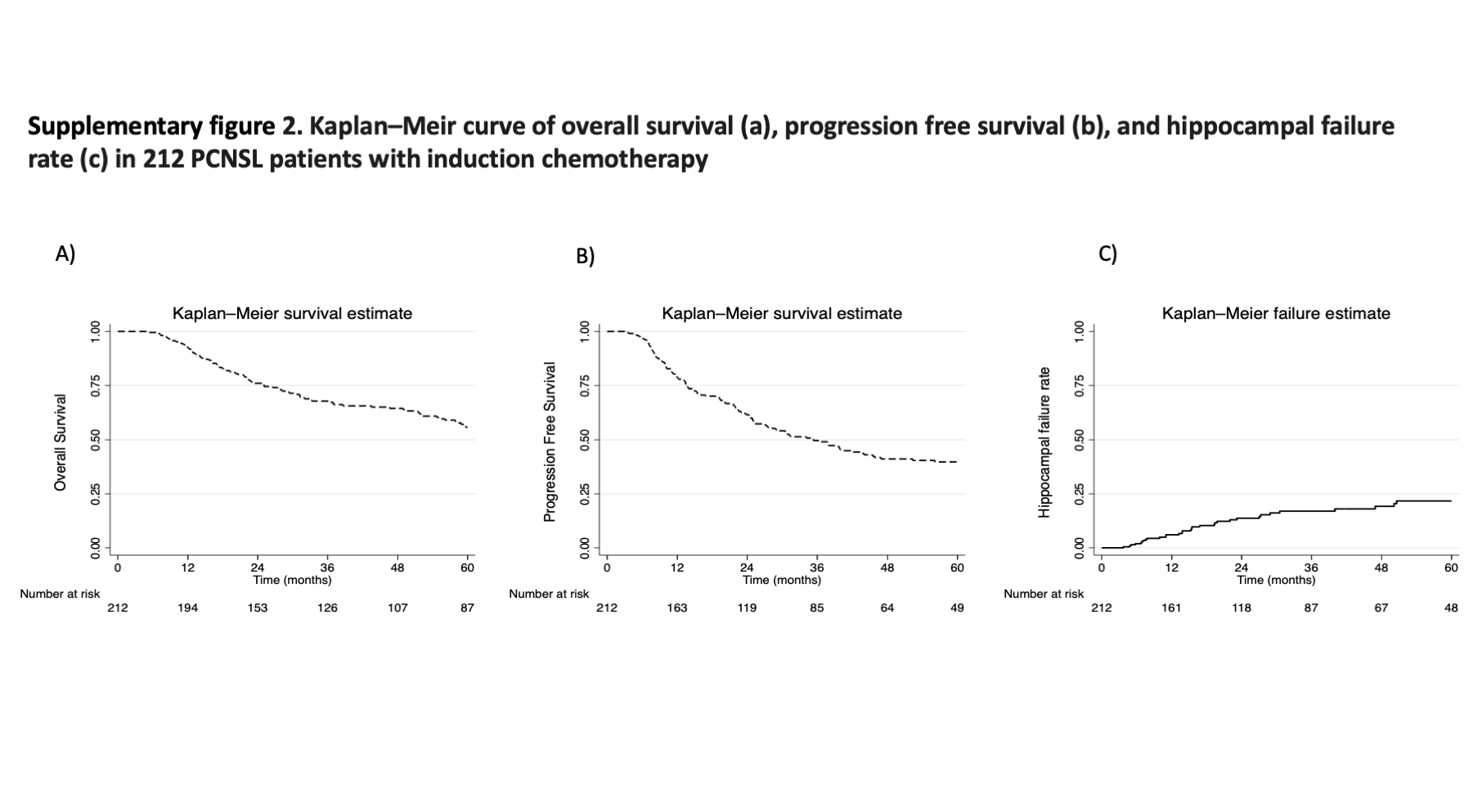

Supplement: Supplementary file 2 — Supplementary file2 (TIFF 5243 KB) Kaplan–Meir curve of overall survival (a), progression free survival (b), and hippocampal failure rate (c) in 212 PCNSL patients with induction chemotherapy [file 11060_2025_4965_MOESM2_ESM.tiff]

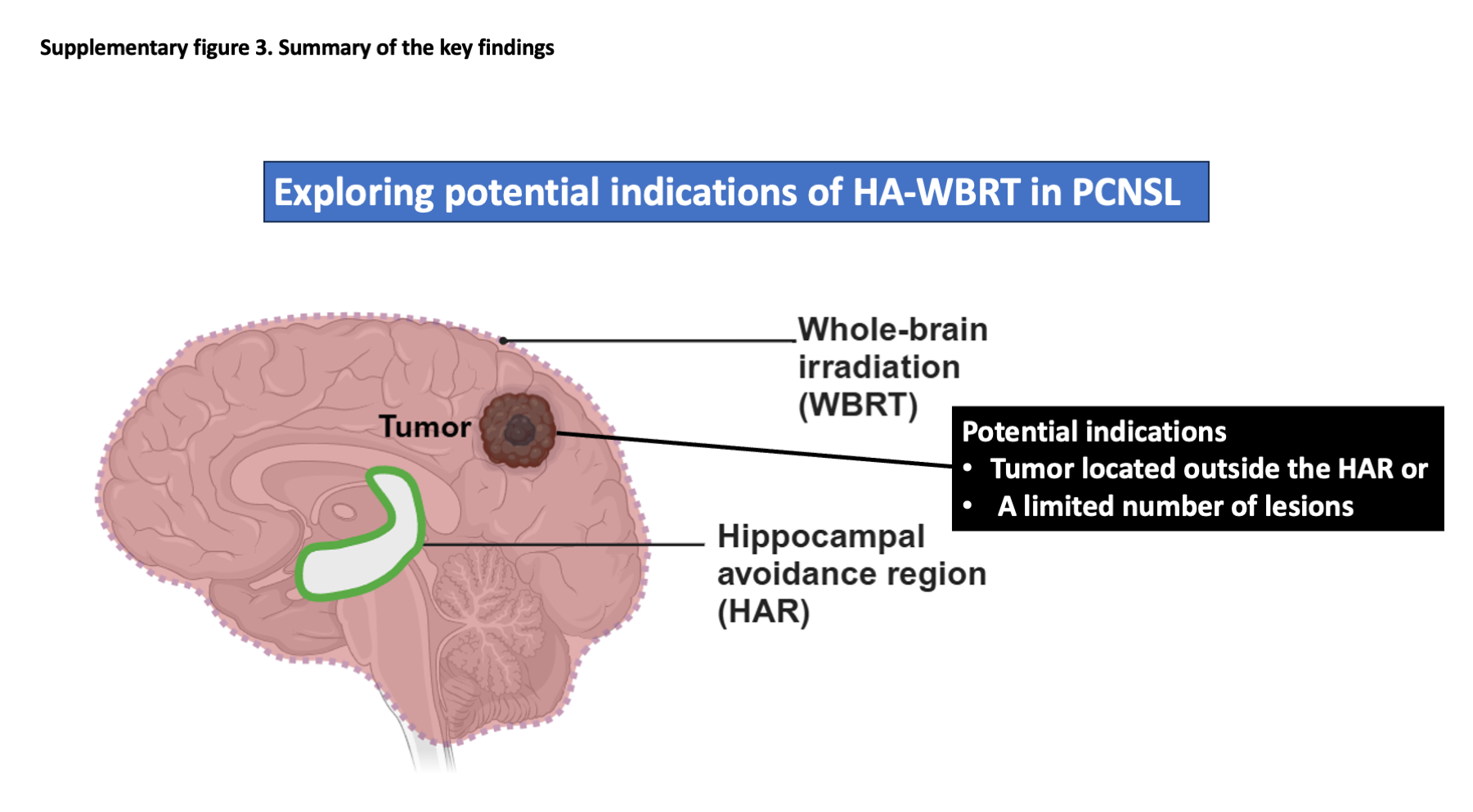

Supplement: Supplementary file 3 — Supplementary file3 (TIFF 5243 KB) Summary of the key findings [file 11060_2025_4965_MOESM3_ESM.tiff]
