# Supplementary material for: Distribution and failure patterns of primary central nervous system lymphoma related to the hippocampus: implications for hippocampal avoidance irradiation
Source: J Neurooncol. 2025 Feb 19;173(1):95–104. doi: 10.1007/s11060-025-04965-7 (PMC12041158; doi:10.1007/s11060-025-04965-7)
Supplement: Supplementary file 4 — Supplementary file4 (DOCX 21 KB) [file 11060_2025_4965_MOESM4_ESM.docx]

**Supplementary table 1. Baseline characteristics of 278 PCNSL diagnosed patients.**

|  | | |
| --- | --- | --- |
| **Characteristics** | **N=278** |  |
| Age at diagnosis (year) | 61 (24-85) |  |
| < 60 | 163 (58.6) |  |
| ≥ 60 | 115 (41.4) |  |
| Sex |  |  |
| Male | 149 (53.6) |  |
| Female | 129 (46.4) |  |
| ECOG performance status |  |  |
| 0–1 | 163 (58.6) |  |
| 2–4 | 115 (41.4) |  |
| Pathology |  |  |
| Diffuse large B-cell lymphoma | 265 (95.3) |  |
| Other B-cell lymphoma | 9 (3.2) |  |
| T-cell lymphoma | 4 (1.4) |  |
| Eye involvement |  |  |
| Yes | 24 (8.6) |  |
| No |  |  |
| CSF involvement |  |  |
| Yes | 27 (9.7) |  |
| No | 251(90.3) |  |
| Diagnosis |  |  |
| Stereotactic biopsy | 243 (84.4) |  |
| Surgery | 46 (15.6) |  |
| Number of lesions |  |  |
| Single | 103 (37.1) |  |
| Multiple | 175 (63.0) |  |
| Deep structure involvement |  |  |
| Yes | 218 (64.8) |  |
| No | 60 (35.2) |  |
|  |  |  |
|  |  |  |

Data are presented as number (%) or median (range).
